# Tetraenone A: A New β-Ionone Derivative from *Tetraena aegyptia*

**DOI:** 10.3390/metabo13121202

**Published:** 2023-12-18

**Authors:** Ahmed Ashour, Asmaa E. Sherif, Selwan M. El-Sayed, Ji-Young Kim, Dae Sik Jang, Abtin Anvari, Abdelbasset A. Farahat, Sabrin R. M. Ibrahim, Gamal A. Mohamed, Bayan E. Ainousah, Raghad F. Aljohani, Razan R. Al-Hejaili, Rahaf H. Khoja, Ahmed H. E. Hassan, Ahmed A. Zaki

**Affiliations:** 1 Department of Pharmacognosy, Faculty of Pharmacy, Prince Sattam Bin Abdulaziz University, Al-Kharj 11942, Saudi Arabia; ae.sherif@psau.edu.sa; 2 Department of Pharmacognosy, Faculty of Pharmacy, Mansoura University, Mansoura 35516, Egypt; ahmed.awad@fulbrightmail.org; 3 Department of Medicinal Chemistry, Faculty of Pharmacy, Mansoura University, Mansoura 35516, Egypt; salwanmahmoud@mans.edu.eg; 4 Department of Life and Nanopharmaceutical Sciences, Kyung Hee University, Seoul 02447, Republic of Korea; k_christina@khu.ac.kr (J.-Y.K.); dsjang@khu.ac.kr (D.S.J.); 5 Master of Pharmaceutical Sciences Program, California Northstate University, 9700 W Taron Dr., Elk Grove, CA 95757, USA; abtin.anvari8815@cnsu.edu (A.A.); abdelbasset.farahat@cnsu.edu (A.A.F.); 6 Department of Pharmaceutical Organic Chemistry, Faculty of Pharmacy, Mansoura University, Mansoura 35516, Egypt; 7 Preparatory Year Program, Department of Chemistry, Batterjee Medical College, Jeddah 21442, Saudi Arabia; sabrin.ibrahim@bmc.edu.sa; 8 Department of Pharmacognosy, Faculty of Pharmacy, Assiut University, Assiut 71526, Egypt; 9 Department of Natural Products and Alternative Medicine, Faculty of Pharmacy, King Abdulaziz University, Jeddah 21589, Saudi Arabia; gahussein@kau.edu.sa; 10 Department of Pharmaceutical Sciences, Faculty of Pharmacy, Umm Al-Qura University, Makkah 21955, Saudi Arabia; baaunosah@uqu.edu.sa; 11 College of Pharmacy, Taibah University, Medina 42353, Saudi Arabia; phraghad0@gmail.com (R.F.A.); alhujilirazan@gmail.com (R.R.A.-H.); ph.rahafkhoja@gmail.com (R.H.K.); 12 Department of Medicinal Chemistry, College of Pharmacy, University of Florida, Gainesville, FL 32610, USA

**Keywords:** megastigmene, tetraenone A, *Tetraena aegyptia*, SARS-CoV-2, drug discovery, life on land, health and wellbeing

## Abstract

In this study, the chemical investigation of *Tetraena aegyptia* (Zygophyllaceae) led to the identification of a new megastigmene derivative, tetraenone A ((2*S*, 5*R*, 6*R*, 7*E*)-2-hydroxy-5,6-dihydro-β-ionone) (**1**), along with (3*S*, 5*R*, 6*S*, 7*E*)-3-hydroxy-5,6-epoxy-5,6-dihydro-β-ionone- (**2**), 3,4-dihydroxy-cinnamyl alcohol-4-glucoside (**3**), 3β,19α-dihydroxy-ursan-28-oic acid (**4**), quinovic acid (**5**), *p*-coumaric acid (**6**), and ferulic acid (**7**), for the first time. The chemical structures of **1**–**7** were confirmed by analysis of their 1D and 2D NMR and HRESIMS spectra and by their comparison with the relevant literature. The absolute configurations of **1** and **2** were assigned based on NOESY interactions and ECD spectra. Conformational analysis showed that **1** existed exclusively in one of the two theoretically possible chair conformers with a predominant *s-trans* configuration for the 3-oxobut-1-en-1-yl group with the ring, while the half-chair conformer had a pseudo-axial hydroxy group that was predominant over the other half-chair conformation. Boat conformations were not among the most stable conformations, and the *s-trans* isomerism was in favor of *s-cis* configuration. In silico investigation revealed that **1** and **2** had more favorable binding interactions with M^pro^ rather than with TMPRSS2. Accordingly, molecular dynamic simulations were performed on the complexes of compounds **1** and **2** with M^pro^ to explore the stability of their interaction with the target protein structure. Compounds **1** and **2** might offer a possible starting point for developing covalent inhibitors of M^pro^ of SARS-CoV-2.

## 1. Introduction

The genus *Tetraena* belongs to the caltrop family (Zygophyllaceae), which is distributed in the deserts and salt marshes of Egypt and is represented by nine species, including *Tetraena aegyptia* [1,2]. It has been used in traditional medicine for a long time to treat various ailments, such as gout and rheumatism [1]. Many studies have documented several biological activities for *Tetraena aegyptia*, such as anti-inflammatory, antidiabetic, and antitumor activities [3,4,5,6,7,8]. Chemical studies on *Tetraena aegyptia* have revealed that quinovic acid and flavonoid glycosides are the main constituents in addition to a sulfonyl epoxy-lignan, which has been recently reported [6,7,8].

**Figure 1:**
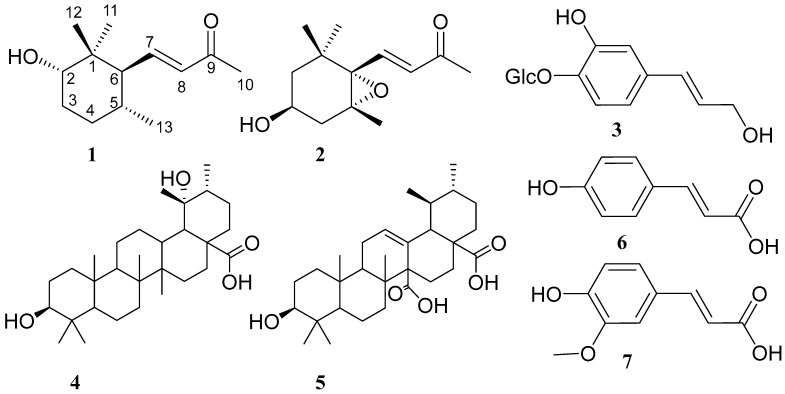
Chemical structures of compounds **1**–**7**.

In the present chemical study on *Tetraena aegyptia*, seven compounds, including two dihydro-β-ionones (**1** and **2**), two triterpenes (**4** and **5**), and three phenolic compounds (**3** and **6**–**7**), were separated (Figure 1) for the first time from the plant. The absolute configurations of **1** and **2** were determined using circular dichroism (CD). CD is a powerful technique used for assigning the stereochemistry of organic compounds. The CD value corresponds to the difference in the absorption of left and right circularly polarized light by a chiral molecule and is expressed in ∆ε (ε^l^ − ε^r^, differential molar extinction coefficient) [9,10]. In this paper, the absolute configurations were obtained by comparing the experimental CD spectra with the theoretical CD spectra, and the conformational analysis was also studied. In 2022, a US patent (US2022/0175868) claimed the usefulness of the extract of the Middle Eastern herb black calla lily (*Arum palaestinum*) as a treatment for COVID-19, which is a viral disease caused by SARS-CoV-2 infection. This patent showed that the extract contains several phytocompounds, including β-ionone epoxide, compound **2**. However, no further information was provided on the possible molecular target. The accumulated research on COVID-19 has demonstrated that two prominent proteins might serve as potential targets to develop possible treatments for COVID-19: the SARS-CoV-2 main protease, which is a viral protein involved in the replication of the virus, and the transmembrane serine protease 2 (TMPRSS2), which is a human protein responsible for viral entry into the host cells and, hence, viral infection. In light of this information, an in silico study of the isolated β-ionone-derived compounds **1** and **2** was conducted against these two molecular targets. M^pro^, a cysteine protease of SARS-CoV-2, is a key target for developing anti-SARS-CoV-2 therapeutics for the treatment of COVID-19. Similar to other cysteine proteases, the active site incorporates catalytic dyad residues, Cys145/His41, which provide a basis for the development of inhibitors. As of September 2021, the pharmaceutical giant Pfizer has initiated phase II/III clinical trials of PF-07321332, an orally administered peptidomimetic SARS-CoV-2 M^pro^ inhibitor, which was later approved as Nirmatrelvir.

## 2. Results and Discussion

### 2.1. Structural Characterization of Compounds ***1*** and ***2***

**Figure 2:**
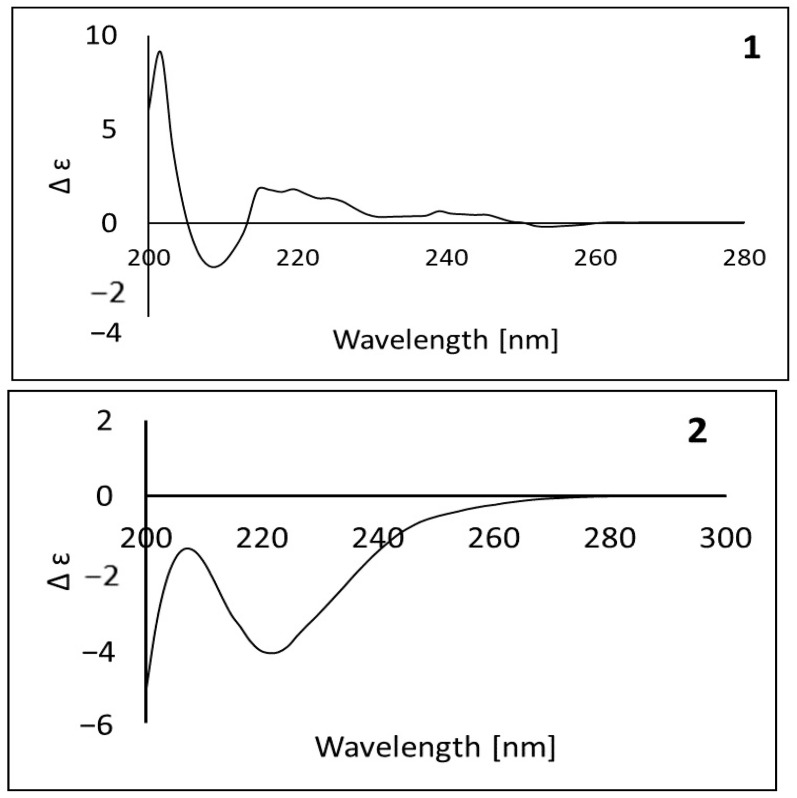
CD spectra of compounds **1** and **2**.

Compound **1** was isolated as a yellow amorphous powder with a molecular formula of C_13_H_22_O_2_ obtained from a [M-H]^−^ ion peak at *m*/*z* 209.15210 (Calcd. 209.15415 for C_13_H_22_O_2_). The ^1^H NMR spectrum possessed signals of three methyl singlets at δ_H_ 2.28, 0.99, and 0.91; one methyl group doublet at δ_H_ 0.86 (*d*, *J* = 6.5 Hz); one oxymethine at δ_H_ 4.53 (*tt*, *J* = 11.6, 4.4 Hz); six aliphatic protons at δ_H_ 1.11 (*m*), 1.31 (*t*, *J* = 12.1 Hz), 1.61 (*t*, *J* = 10.6 Hz), 1.79 (*m*), 2.02 (*m*), and 2.31 (*dd*, *J* = 9.6, 5.2 Hz); and a pair of conjugated olefinic protons at δ_H_ 6.69 (*dd*, *J* = 15.9, 10.3 Hz) and 6.11 (*d*, *J* = 15.9 Hz). The ^13^C and DEPT NMR spectra of **1** showed thirteen carbon signals, including four methyls, two methylenes, three methines, two olefinic carbons, one quaternary carbon, and one carbonyl. All protons were connected to the corresponding carbons based on the observed correlations in the HMQC spectrum. The coupling pattern of the olefinic protons at δ_H_ 6.69 (*dd*, *J* = 15.9, 10.3 Hz) and 6.1 (*d*, *J* = 15.9 Hz) and a methyl singlet at δ_H_ 2.28 connected to the carbonyl group at δ_C_ 200.8 indicated the existence of a *trans*-disubstituted double bond, characteristic for a 7*E*-buten-2-one side chain [11,12,13]. The two-dimensional NMR correlations (^1^H-^1^H COSY and HMBC) enabled the full ^13^C assignment, and the structure of 3-hydroxy-5,6-dihydro-β-ionone was concluded. The relative configuration at C-2, C-5, and C-6 was established based on extensive analysis of the coupling patterns of the cyclohexane ring protons and NOE interactions. The two methyl singlets at δ_H_ 0.91 and 0.99 are connected to C-1 and were assigned to C-12 and C-11, respectively. The NOE correlation of the singlet at δ_H_ 0.91 with H-7 indicated the β-orientation of CH_3_-12. The oxymethine proton at δ 4.53 (*tt*, *J* = 11.6, 4.4 Hz, H-2) showed NOE correlations with CH_3_-12 (δ_H_ 0.91), H-4β (δ_H_ 2.02), H-3β (δ_H_ 2.31), and H-5β (δ_H_ 1.79), indicating the α-orientation of 2-OH. Furthermore, the α-orientation of CH_3_-13 was concluded from the correlations of H-5 with H-7 (6.69, *dd*, *J* = 15.9, 10.3 Hz) and CH_3_-12 in the NOESY spectrum. The β-position of the buten-2-one side chain at C-6 was confirmed by the observed NOE correlations of H-6 with CH_3_-11 and CH_3_-13. The relative configuration at C-2, 5, and 6 was determined to be 2α-OH, 5α-CH_3,_ and 6β-buten-2-one. The CD spectrum of **1** (Figure 2) showed positive Cotton effects at ∆ε_201_._6 nm_ + 9.108 and ∆ε_219_._5 nm_ + 1.805 and a negative Cotton effect at ∆ε_208_._13 nm_ −2.28. The structure of compound **1** was established as (2*S*, 5*R*, 6*R*,7*E*)-3-hydroxy-7-megastigmene-9-one) and named tetraenone A.

Compound **2** was obtained as a yellowish-white amorphous powder with a molecular formula of C_13_H_20_O_3_ deduced from a [M + Na]^+^ ion peak at *m*/*z* 247.1308 (Calcd. 247.1305 for C_13_H_20_O_3_ Na). The inspection of the ^13^C NMR spectrum indicated the presence of thirteen carbon signals, suggesting **2** possesses the same β-ionone skeleton as that of **1**. The ^1^H NMR spectrum showed four methyl singlets at δ_H_ 2.30, 0.98, 1.25, and 1.19; four aliphatic protons at δ_H_ 1.48 (*dd*, *J* = 13.1, 9.9 Hz), 1.79 (*d*, *J* = 12.4 Hz), 1.96 (*dd*, *J* = 14.8, 7.8 Hz), and 2.50 (*dd*, *J* = 14.8, 5.2 Hz); two olefinic protons at δ_H_ 6.20 (*d*, *J* = 15.8 Hz) and 7.17 (*d*, *J* = 15.8 Hz); and one oxymethine proton at δ_H_ 4.51 (*m*). The ^13^C NMR spectrum showed the presence of four methyls, two methylenes, two olefinic carbons, one methine, three quaternary carbons, and one carbonyl group. The absence of signals assigned to H-5 and H-6, as well as the multiplicity of H-7 (7.17, *d*, *J* = 15.8 Hz), indicated the oxygenation of C-5 and C-6. The up-field shift of C-5 and C-6 at δ_C_ 67.5 and 70.4, respectively, together with HRESIMS revealed the presence of a 5,6-epoxy ring. The third oxymethine carbon at δ_C_ 72.4 in the ^13^C NMR spectrum was attributed to C-3. The 7*E*-buten-2-one side chain was confirmed by the coupling of H-7 and H-8 (*d*, *J* = 15.8 Hz) [13]. The stereochemistry at C-3, C-5, and C-6 was specified by the analysis of the NOESY spectrum. The observed NOE correlations of CH_3_-13 (1.19, *s*) with H-8, H-7, H-4β (1.96), and CH_3_-12 indicated the *trans*-5,6-epoxide. The NOE correlations of H-3 with CH_3_-11, H-2α (δ_H_ 1.79), and H-4α (δ_H_ 2.50) revealed the β-orientation of the C-3 hydroxyl group. Thus, the relative configuration of **2** was determined to be 3β-OH, 5β-CH_3_, and 6β-buten-2-one. The *trans*- and *cis*-epoxides of compound **2** were previously synthesized by Mori in 1974, and the stereochemistry of *trans*-epoxide was determined as 3*S*, 5*R*, 6*S* [14,15,16,17,18]. Further, this absolute configuration w*as* concluded from a negative Cotton effect at 232 nm in the CD spectrum. The CD spectrum of **2** (Figure 2) revealed the presence of a negative Cotton effect at ∆ε_221_._13 nm_ − 4.12, which is in full accordance with the 3*S*, 5*R*, 6*S* configuration. The structure of compound **2** was determined to be (3*S*, 5*R*, 6*S*, 7*E*)-5,6-epoxy-3-hydroxy-7-megastigmene-9-one [16,17,18]. It is noteworthy that **2** was previously isolated from *Cestrum parqui*. Based on the literature, this is the first report on the detailed absolute configuration and conformational analysis of **2**.

In addition, 3,4-dihydroxy-cinnamyl alcohol-4-glucoside (demethyl coniferin) (**3**) [19,20], 3β,19α-dihydroxy-ursan-28-oic acid (**4**) [21], quinovic acid (**5**) [22,23], *p*-coumaric acid (**6**), and ferulic acid (**7**) [24] were isolated and identified based on the spectroscopic analysis and comparison with published data.

### 2.2. Conformational Analysis of Compounds ***1*** and ***2***

Conformational analyses of the forms of compounds **1** and **2** were conducted by adopting the systematic conformational search method using the Merk molecular force field (MMFF). The results are shown in the following sections.

#### 2.2.1. Conformational Analysis of Compound **1**

Compound **1** has a 1,1-dimethylcyclohexane scaffold bearing a 3-oxobut-1-en-1-yl group at the 6-position in a *trans* relationship with the hydroxy group at the 2-position and the methyl group at the 5-position. Conformational analysis showed two major conformers that constitute more than 70% of the calculated conformers’ population (0.705 cumulative Boltzmann weight) and, together with the other three conformers, constitute more than 95% of the calculated conformers’ population (0.959 cumulative Boltzmann weight). Theoretically, the six-membered cyclohexane ring can show two minima, namely chair and twist-boat conformations, and two maxima, namely boat and half-chair conformations. However, the conformational analysis showed that the five most energetically favorable conformers for compound **1** exclusively adopt chair conformations (relative energy difference within 7.17 kJ/mol). In addition, the hydroxy group at the 2-position, the 3-oxobut-1-en-1-yl group at the 6-position, and the methyl group at the 5-position of all the five conformers are exclusively in axial, equatorial, and equatorial positions, respectively. This indicates the absence of ring flipping to accommodate the other theoretically possible chair conformation in which the hydroxy group, the 3-oxobut-1-en-1-yl, and the methyl group at 5-position might accommodate equatorial, axial, and axial positions, respectively. Accordingly, the different conformers obtained arose from the different *s-cis* and *s-trans* conformations arising from rotation around sigma-bonds in the 3-oxobut-1-en-1-yl group as well as the position of the hydroxyl group’s hydrogen atom. Interestingly, the *s-trans* relationship between the olefinic double bond and the cyclohexane ring was predominant in the most energetically favorable four conformers, constituting 93.6% of the conformers’ population. Meanwhile, the *s-cis* relationship between the olefinic double bond and the cyclohexane ring existed in only the fifth most energetically favorable conformer, which constitutes only 2.3% of the conformers’ populations. In contrast to the strong preference of the *s-trans* conformer considering the olefinic double bond and the cyclohexane ring, the *s-cis*/*trans* isomerization of the olefinic double bond and the carbonyl double bond was less impactful and was encountered with almost equal probabilities in the identified most energetically favorable conformers. On the other side, the position of the hydrogen atom of the hydroxyl group was clearly found in the most energetically favorable five conformers. This was indicated by three-quarters of the conformers’ population having an almost 180° dihedral angle (i.e., antiperiplanar), involving H–O–C^2^–C^1^ atoms (conformers 1, 2, and 5), while the dihedral angle was almost 80° (i.e., synclinal) in the remaining one-quarter of the conformers’ population (Table 1).

#### 2.2.2. Conformational Analysis of Compound **2**

Compound **2** has a cyclohexene oxide (AKA 7-oxabicyclo [4.1.0]heptane) scaffold bearing geminal dimethyl substituents at the 1-position as well as a 3-oxobut-1-en-1-yl group at the 6-position in a *cis* relationship with the hydroxy group at the 3-position and the methyl group at the 5-position. Accordingly, the oxide group would be *trans*-configured to the 3-oxobut-1-en-1-yl, hydroxy, and 5-methyl groups. In contrast to compound **1**’s cyclohexane scaffold, which showed a limited number of conformers, the condensation of the small oxirane ring had pronounced effects on the conformational properties of the six-membered ring, resulting in a significantly larger number of conformers for compound **2**. Referring to the six-membered cyclohexane ring, two interconverting half-chair conformations are theoretically possible in addition to *endo*/*exo*-boat conformations [25]. Despite the previous studies suggesting the cyclohexene oxide to have boat conformations in equilibria with the most stable half-chair conformations [25,26], only half-chair conformations could be detected in the retrieved twelve most energetically favorable conformers (relative energy difference within 7.50 kJ/mol). Together, these twelve conformers constitute 96.3% of the conformers’ population (cumulative Boltzmann weights of 0.963). However, 52.0% of the conformers’ population exists in the form of only two conformers (conformers 1 and 2). Notably, all of the retrieved twelve conformers accommodate the 3-oxobut-1-en-1-yl, and the 5-methyl groups in pseudo-equatorial positions. Meanwhile, the hydroxyl group accommodated a pseudo-axial in nine conformers constituting 81.5% of the conformers’ population (combined Boltzmann weights of 0.815 for conformers 1–3 and 6–11) and pseudo-equatorial in only three conformers constituting 14.8% of the conformers’ population (combined Boltzmann weights of 0.148 for conformers 4, 5, and 12) [27,28].

This indicates that ring flipping equilibrium is more in favor of the half-chair conformation having the hydroxy group as pseudo-axial. In comparison with compound **1**, the position of the hydrogen of the hydroxyl group was less influential, showing 60% of the retrieved conformers (1, 3, 4, 7, and 8) accommodating antiperiplanar/synclinal dihedral angles (almost 180/60°) for H–O–C^3^–C^4^ and H–O–C^3^–C^2^–atoms, respectively, and 39% of the retrieved conformers (2, 5, 6, 10, and 11) have the antiperiplanar/synclinal dihedral angles (almost 180/60°) for H–O–C^3^–C^2^ and H–O–C^3^–C^4^–atoms, respectively. While the *s-cis*/*trans* relationship (arising from rotation around sigma-bonds in the 3-oxobut-1-en-1-yl group) between the olefinic double bond and the ring was much more influential than the *s-cis*/*trans* relationship between the olefinic and the carbonyl double bonds, both have a potential impact in compound **2**. Thus, at least 83.2% of the conformers’ population (conformers 1, 2, 3, 4, 5, 6, 9, and 12) have the olefinic double bond and the oxirane ring in *s-cis* relationship compared with at least 13.1% of the conformers’ population (conformers 7, 8, 10, and 11) having them in *s-trans* relationship. Meanwhile, at least 75.9% of the conformers’ population (conformers 1, 2, 4, 5, 7, 9, 11, and 12) have the olefinic and carbonyl double bonds in *s-cis* relationship compared with at least 20.4% of the conformers’ population (conformers 3, 6, 8, and 10) having them in *s-trans* relationship. Together, these results indicate the preference of compound **2** to accommodate the half-chair conformation in which the hydroxyl group accommodates the pseudo-axial position and has *s-cis* relationships for the olefinic double bond/oxirane ring and the olefinic/carbonyl double bonds (Table 2).

#### 2.2.3. Assignment of the Absolute Stereochemical Configuration of Compounds **1** and **2** (ECD Spectra)

Electronic circular dichroism (ECD) spectra of (*R*)- and (*S*)-enantiomers of compounds **1** and **2** were calculated to assign their absolute configurations. As ab initio calculations are computationally demanding, a proper compromise between computational cost and accuracy is needed. Consequently, the time-dependent density functional theory (TDDFT) method was implemented for its satisfactory accuracy/computational load. The implementation of a proper combination of functional/basis is pivotal for TDDFT calculations. Benchmarking studies have indicated that the calculation of ECD spectra using range-separated hybrid functions such as CAM-B3LYP is more accurate than only hybrid functions such as B3LYP. Benchmarking studies also indicated more reliability for the implementation of Ahlrichs basis sets such as SVP for TDDFT calculations rather than split-valence Pople basis sets such as 6-31+G. Therefore, CAM-B3LYP/SVP as a functional/basis set combination was employed in calculating ECD spectra using a conductor-like polarizable continuum model (CPCM) as a solvent model.

**Figure 3:**
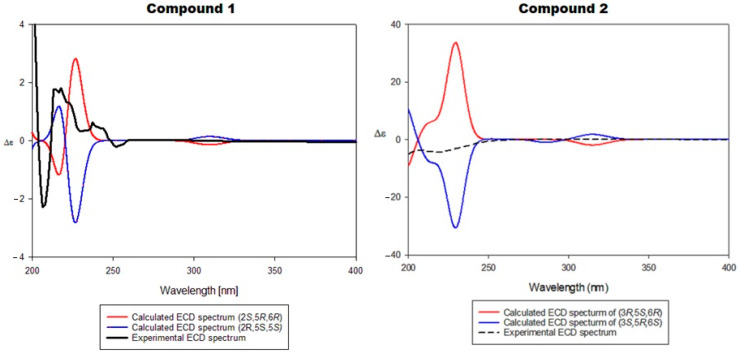
CD and ECD spectra of compounds **1** and **2**.

The experimental CD spectrum of compound **1** (Figure 3) showed a positive Cotton effect at 219.5 nm and a negative Cotton effect at 208.13 nm. This experimental CD spectrum matched the calculated ECD spectrum for the 2*S*, 5*R*, 6*R* stereoisomer; thus, compound **1** was established as (2*S*, 5*R*, 6*R*, 7*E*)-2-hydroxy-5,6-dihydro-β-ionone. Meanwhile, the experimental CD spectrum of compound **2** showed a negative Cotton effect at 221.13 nm, which matched the calculated ECD spectrum for the 3*R*, 5*S*, 6*R* stereoisomer; thus, the absolute configuration of compound **2** was concluded as (3*S*, 5*R*, 6*S*, 7*E*)-3-hydroxy-5,6-epoxy-5,6-dihydro-β-ionone (also named as (3*S*, 5*R*, 6*S*, 7*E*)-5,6-epoxy-3-hydroxy-7-megastigmene-9-one).

### 2.3. In Silico Evaluation of Bioactivity of Compounds ***1*** and ***2***

#### 2.3.1. In Silico Evaluation of Compounds **1** and **2** against SARS-CoV-2 Main Protease

**Figure 4:**
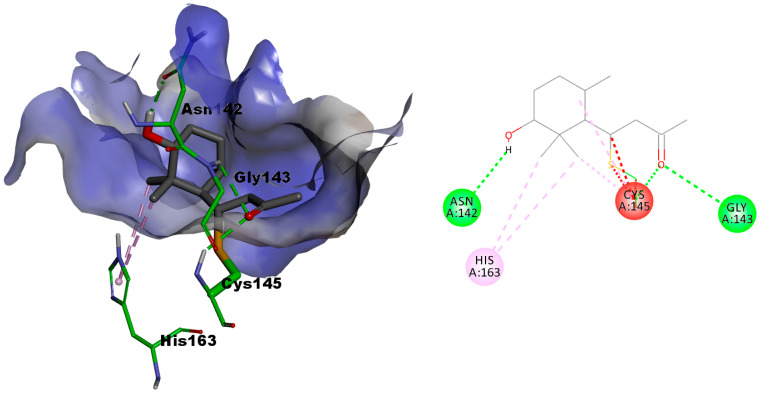
Compound **1** covalently docked into the active site of M^pro^ of SARS-CoV-2.

As a validated drug target, developing the small molecule inhibitors of M^pro^ rather than the peptidomimetic inhibitor might offer multiple advantages in the fight against COVID-19. As is clear from the crystal structure of M^pro^ (PDB: 6lu7), the nucleophilic key residue Cys145 can establish a covalent interaction with inhibitors compromising an electrophilic moiety. Considering that the structures of compounds **1** and **2** incorporate an α,β-unsaturated carbonyl moiety, which is a well-known electrophilic moiety that might undergo nucleophilic addition, it was interesting to explore whether these compounds can bind to M^pro^ and establish a covalent interaction with Cys145. Therefore, in silico covalent docking was addressed to predict the abilities of compounds **1** and **2** to bind and inhibit M^pro^ of SARS-CoV-2. As shown in Figure 4, compound **1** could successfully dock into the active site of M^pro^ of SARS-CoV-2 with a calculated favorable docking score of −4.89957 kcal/mol.

**Figure 5:**
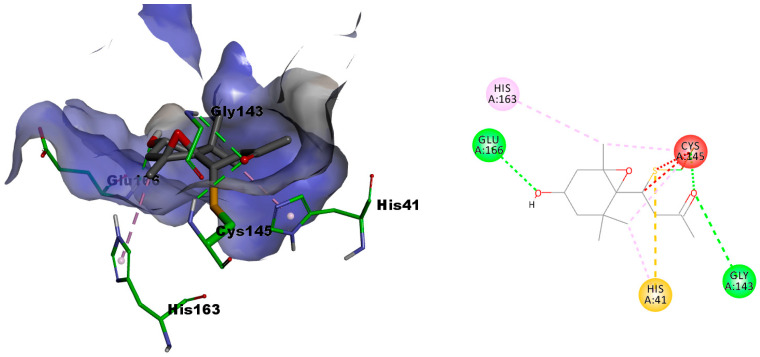
Compound **2** covalently docked into the active site of M^pro^ of SARS-CoV-2.

In addition to establishing a covalent interaction with the catalytic cys145, it established a network of favorable interactions involving two hydrogen-donor bonding interactions between the compound **1** oxygen atom of the carbonyl group and the backbone NH of both of Cys145 and Gly143 and one hydrogen bond acceptor between the compound **1** OH group and the side chain carbonyl group of Asn142, as well as two hydrophobic π-alkyl interactions between both vicinal methyl groups of compound **1** and the imidazole ring of His163. Compound **2**, as shown in Figure 5, also docked successfully into the active site of M^pro^ of SARS-CoV-2 with an almost similar docking score to compound **1** (docking score of −4.85742 kcal/mol for compound **2** versus docking score of −4.89957 kcal/mol for compound **1**). As illustrated in Figure 2, the oxygen atom of the carbonyl group of **2** still maintains the two hydrogen donor bonding interactions with the backbone NH of both Cys145 and Gly143.

However, only one methyl group of the two vicinal methyl groups of **2** is in a hydrophobic π-alkyl interaction with the imidazole ring of His41, while the methyl group vicinal to the epoxy moiety is in a hydrophobic π-alkyl interaction with imidazole ring of His163. Meanwhile, the OH group of **2** is involved in a hydrogen-bond donor interaction with the backbone NH of Glu166. The epoxy group of **2** did not contribute any significant binding interactions, and hence, it might be insignificant for activity.

#### 2.3.2. In Silico Evaluation of Compounds **1** and **2** against Transmembrane Serine Protease 2 (TMPRSS2)

**Figure 6:**
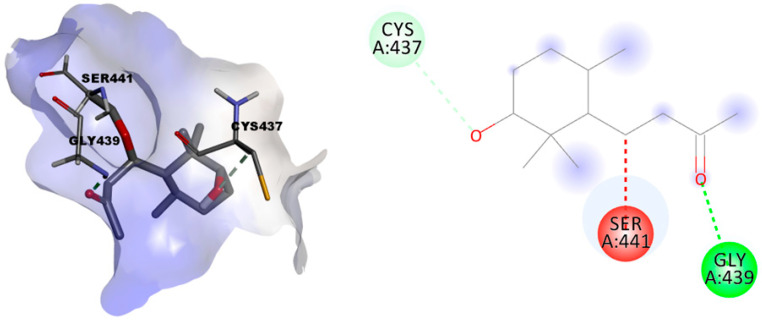
Compound **1** covalently docked into the active site of TMPRSS2.

TMPRSS2 is a member of the serine protease family that is highly expressed in nasal, bronchial, and gastrointestinal epithelial cells. It was found that TMPRSS2 is a key factor involved in viral entry and spread inside the human body. SARS-CoV-2 utilizes this enzyme to cleave its spike protein (S protein) and prime the virus for cell entry. Inhibitors of TMPRSS2 are expected to be promising treatments for SARS-CoV-2 infections. The substrate site of TMPRSS2 encompasses a catalytic triad that involves the amino acid residues Ser441, Asp435, and His296. As found in the crystal structure of TMPRSS2 (PDB ID: 7meq), covalent inhibitors undergo nucleophilic attack by Ser441 to establish a covalent bond, resulting in inactivation and inhibition of TMPRSS2. As mentioned above, the structures of compounds **1** and **2** incorporate an α,β-unsaturated carbonyl moiety, which is a well-known electrophilic moiety that might undergo nucleophilic addition. Accordingly, a covalent docking was carried out to explore the ability of compounds **1** and **2** to dock into the active site of TMPRSS2 and establish stable bonding interactions with it. Both compounds **1** and **2** docked successfully with docking scores of −4.1051 and −3.8134 kcal/mol, respectively. As illustrated in Figure 6, compound **1** was able to successfully form a covalent bonding interaction with Ser441.

**Figure 7:**
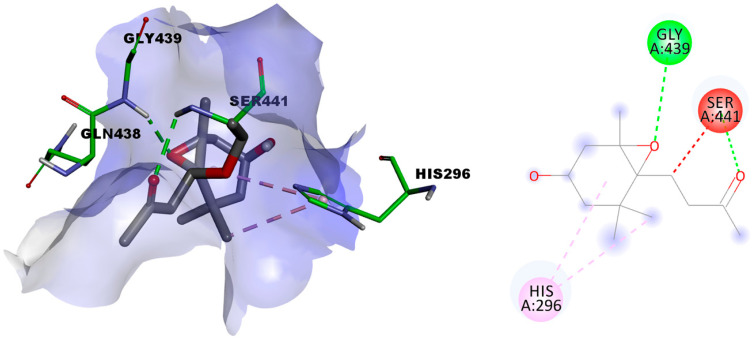
Compound **2** covalently docked into the active site of TMPRSS2.

In addition, it formed a favorable hydrogen-bonding acceptor interaction between the oxygen atom of the carbonyl moiety and the backbone NH of the Gly439 residue. Also, it formed a carbon–hydrogen interaction between the OH group and Cys437. Regarding compound **2**, successful covalent docking was also established into the active site of the enzyme as shown in Figure 7, with a maintained covalent bond with Ser441 via its α,β-unsaturated carbonyl warhead moiety.

Additionally, it formed other favorable interactions with other amino acid residues inside the active site, where a hydrogen-bond donor interaction was formed between the oxygen atom of the carbonyl moiety, as in compound **1**, but here with Ser441 of the catalytic triad of the active site. Moreover, the epoxy group of **2** contributed successfully to a hydrogen-bond donor interaction with Gly439. This interaction suggested that the epoxy functionality was of interesting value, regarding the interaction of compound **2**, in contrast to compound **1**. Furthermore, two favorable hydrophobic π-alkyl interactions were formed between His296 of the catalytic triad of the enzyme with each cyclohexane ring and one methyl group of the two vicinal methyl groups of **2**.

#### 2.3.3. Molecular Dynamic Simulations

**Figure 8:**
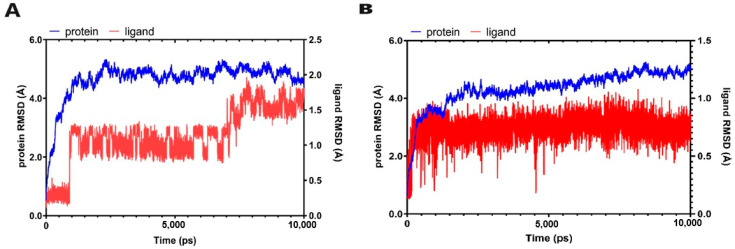
The ligand–protein RMSD plot of compounds **1** and **2** complexed with M^pro^ of SARS-CoV2: (**A**) compound **1**; (**B**) compound **2**. Left and right Y-axes represent the RMSD values of protein and ligand in molecular distance unit Angstrom (Å). X-axis demonstrates the time in picoseconds (ps).

**Figure 9:**
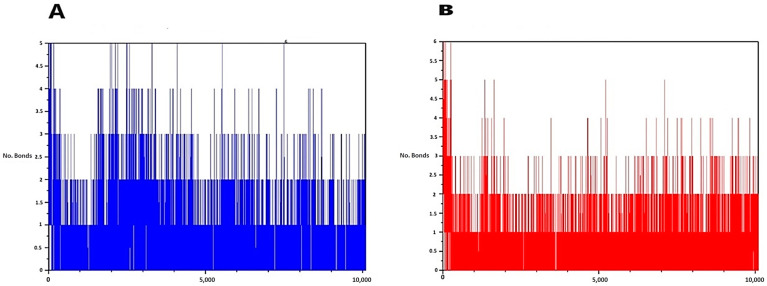
The timeline plot of hydrogen bonding interactions between compounds **1** and **2** and M^pro^ of SARS-CoV2: (**A**) compound **1**; (**B**) compound **2**. Y-axis represents the number of hydrogen bonds, and X-axis demonstrates the time in picoseconds (ps).

A molecular dynamic simulation was performed to evaluate the stability of target–ligand complexes for compounds **1** and **2** with their target protein. By examination of the docking scores of compounds **1** and **2**, it was found that their docking scores with M^pro^ were better than those in the case of docking with the TMPRSS2 enzyme. Accordingly, the best docking pose for each compound with M^pro^ was subjected to molecular dynamic simulation over a period of 10 ns. The whole ligand–protein complex was utilized to evaluate the stability of the complex formed during the docking experiment. Regarding compound **1**, the simulation revealed that there was an initial increase in the root mean square deviation (RMSD) value for the protein backbone until it reached about 4.5–5 Å in a period of about 2 ns (Figure 8). The RMSD value regarding the ligand of the same complex showed initial fluctuation until about 8 ns, and then it was slightly stabilized for the rest of the simulation time. Similarly, compound **2** was found to have almost the same pattern (Figure 8). The average number of hydrogen bonds formed between the ligand and target protein was calculated and plotted against time frames. Again, compounds **1** and **2** were found to form about the same average number of hydrogen bonds (Figure 9). Collectively, molecular dynamic simulation studies have revealed that both investigated compounds reach stability at about the same time, and they are able to form nearly the same average number of hydrogen bonds with their target, M^pro^ of SARS-CoV2.

## 3. Materials and Methods

### 3.1. Plant Material

Aerial parts of *T. aegyptia* were collected from the coastal desert in Egypt, and a voucher specimen (ZA-35-PD) was kept in the Department of Pharmacognosy, Faculty of Pharmacy, Mansoura University.

### 3.2. Extraction and Isolation

**Scheme 1:**
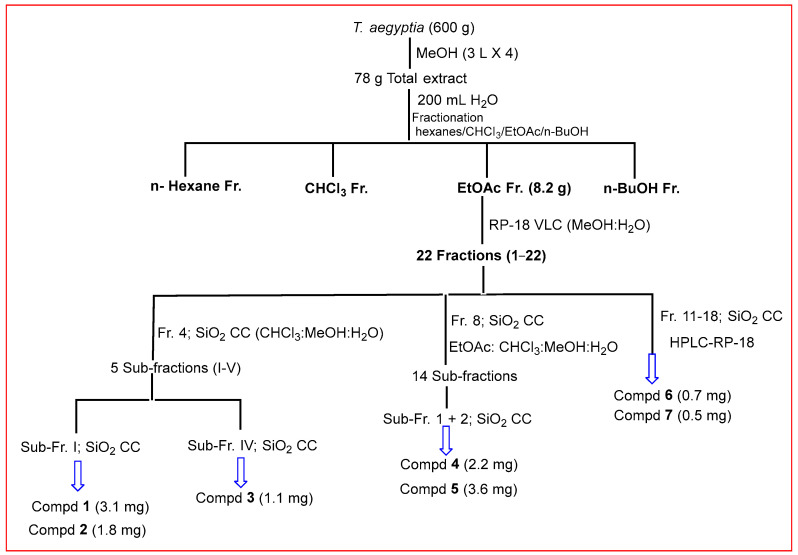
Extraction of *T. aegyptia* and isolation of compounds **1**–**7**.

The air-dried aerial parts of *T. aegyptia* (600 g) were exhaustively extracted with methanol at room temperature (3 L × 4). The dried crude extract (78 g) was obtained by evaporating the methanol extract under reduced pressure at 45 °C (Scheme 1). The total extract was suspended in 200 mL of distilled water and fractionated with hexanes, CHCl_3_, EtOAc, and n-butanol. The EtOAc extract (8.2 g) was applied to a VLC of RP-18 silica (20 cm × 4 cm) and eluted with 1 L of the following gradients: MeOH:H_2_O (1:9, 2:8, 3:7, 4:6, 5:5, 6:4, 7:3, 8:2, 9:1, and 10:0) to obtain 22 fractions (1–22). Fraction 4 (160 mg) was subjected to silica gel column chromatography (100 cm × 2 cm), using CHCl_3_:MeOH:H_2_O (32:8:1 and 30:10:1) to obtain five sub-fractions (I-V). Sub-fraction II (45.2 mg) was re-chromatographed on a silica gel and eluted with CHCl_3_:MeOH:H_2_O (32:8:1 and 30:10:1) to obtain compounds **1** (3.1 mg) and **2** (1.8 mg). Sub-fraction IV (76 mg) was applied on silica gel CC (SiO_2_ CC) using CHCl_3_:MeOH:H_2_O (32:8:1) as an eluent to afford compound **3** (1.1 mg). Fraction 8 (780 mg) was subjected to silica gel CC (100 cm × 3.0 cm) and eluted with EtOAc:CHCl_3_:MeOH:H_2_O mixtures (15:8:4:1, 10:6:4:1, and 6:4:4:1) to obtain 14 sub-fractions. Sub-fractions 1 (60 mg) and 2 (51 mg) were purified through silica CC using EtOAc:CHCl_3_:MeOH:H_2_O mixtures (15:8:4:1, 10:6:4:1, and 6:4:4:1) to yield compounds **4** (2.2 mg, obtained in a mixture with compound **1**) and **5** (3.6 mg). Fractions 11–18 (5 mg) were purified using reversed-phase HPLC (Waters Alliance 2795, equipped with photodiode array detector, and Luna C18 column (150 × 4.6 mm, 5 μm particle size; Phenomenex, Inc., Torrance, CA, USA)), using acetonitrile 0.1% FA (A) and water with 0.1% FA (B) in a gradient mode: A/B 25/75 for 5 min, A/B 35/65 for 15 min, and A/B 45/55 for the next 20 min at a rate of 1 mL/min. The response was detected at 254 nm to obtain compounds **6** (0.7 mg, R_t_ 25.080) and **7** (0.5 mg, R_t_ 26.176).

### 3.3. Conformational Analysis and Electronic Circular Dichroism (ECD) Spectra

Three-dimensional models of compounds **1** and **2** were generated using Chem3D. Spartan14 software (Wavefunction, Inc., Irvin, CA, USA; 2014) was used for conformational analysis using the systematic stepped method. MMFF was employed as the force field. The conformers outside an energy window of 40 kJ mol^−1^ above the energy of the global minimum conformation were excluded. Conformers were optimized using Gaussian 09 software (B3LYP/6-31+G(d,p)). TDDFT ECD spectra were calculated using CAM-B3LYP/SVP and CPCM, employing MeOH as a solvent model.

### 3.4. In Silico Study

Structures of compounds **1** and **2** were sketched, and their energy was minimized. Crystal structures of M^pro^ (PDB: 6lu7) and TMPRSS2 (PDB ID: 7meq) were retrieved from the protein data bank and prepared. The covalent docking to the active site was performed following the known protocols defining Cys145 or Ser441 as the reactive site in a Michael addition reaction for M^pro^ or TMPRSS2, respectively. Binding scores were calculated and refined using the GBVI/WSA dG force field. The best five refined poses were retrieved, visualized, and analyzed. Molecular dynamic simulations were performed using NAMD, employing docked complexes of compound **1** or **2** with M^pro^.

## 4. Conclusions

The chromatographic investigation of *Tetraena aegyptia* methanol extract revealed the presence of seven compounds (**1**–**7**) isolated for the first time from the plant. The stereochemistry of compounds **1** and **2** was deduced from 2D NMR and CD analyses, as well as ECD calculations. Conformational analysis of compound **1** showed the existence of only one of the two theoretically possible chair conformers, suggesting the absence of ring flipping. This demonstrated different conformers arising from the different *s-cis* and *s-trans* isomerism from the rotation around sigma-bonds of the 3-oxobut-1-en-1-yl group with a predominant *s-trans* relationship. Meanwhile, conformational analysis of compound **2** showed the absence of boat conformations from the most stable conformations while the half-chair conformer having a pseudo-axial hydroxy group was predominant over the other half-chair conformation and the *s-cis* and *s-trans* isomerism was in favor of the *s-cis* relationship. In silico investigation of possible covalent inhibitors of M^pro^ of SARS-CoV-2 and TMPRSS2 showed that compounds **1** and **2** have more favorable binding interactions with M^pro^ than with TMPRSS2 and might offer a possible starting point to develop covalent inhibitors of M^pro^ of SARS-CoV-2. These results were ascertained via the molecular dynamic simulation study. The isolated compounds will expand the metabolic profile of *Tetraena aegyptia* to include other natural products and may also help to explain and expect the pharmacological activities of the plant.

## Figures and Tables

**Table 1:** Calculated conformers of compound **1** that constitute more than 95% of the conformations’ population.

Conf. No.	Relative Energy (kJ/mol)	Boltzmann Weights	Cumulative Boltzmann Weights	Compound 1 (α-OH)	Compound 1 (β-OH)
				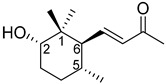	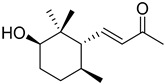
1	0.00	0.406	0.406	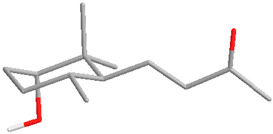	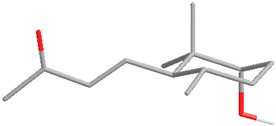
2	0.75	0.300	0.705	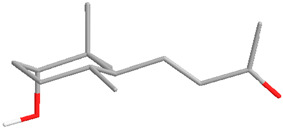	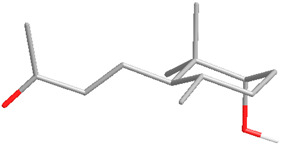
3	3.07	0.117	0.823	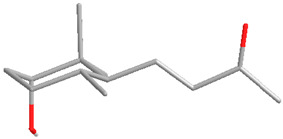	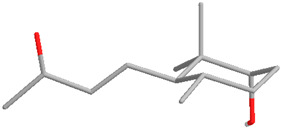
4	3.16	0.114	0.936	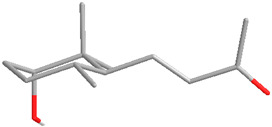	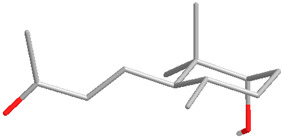
5	7.17	0.023	0.959	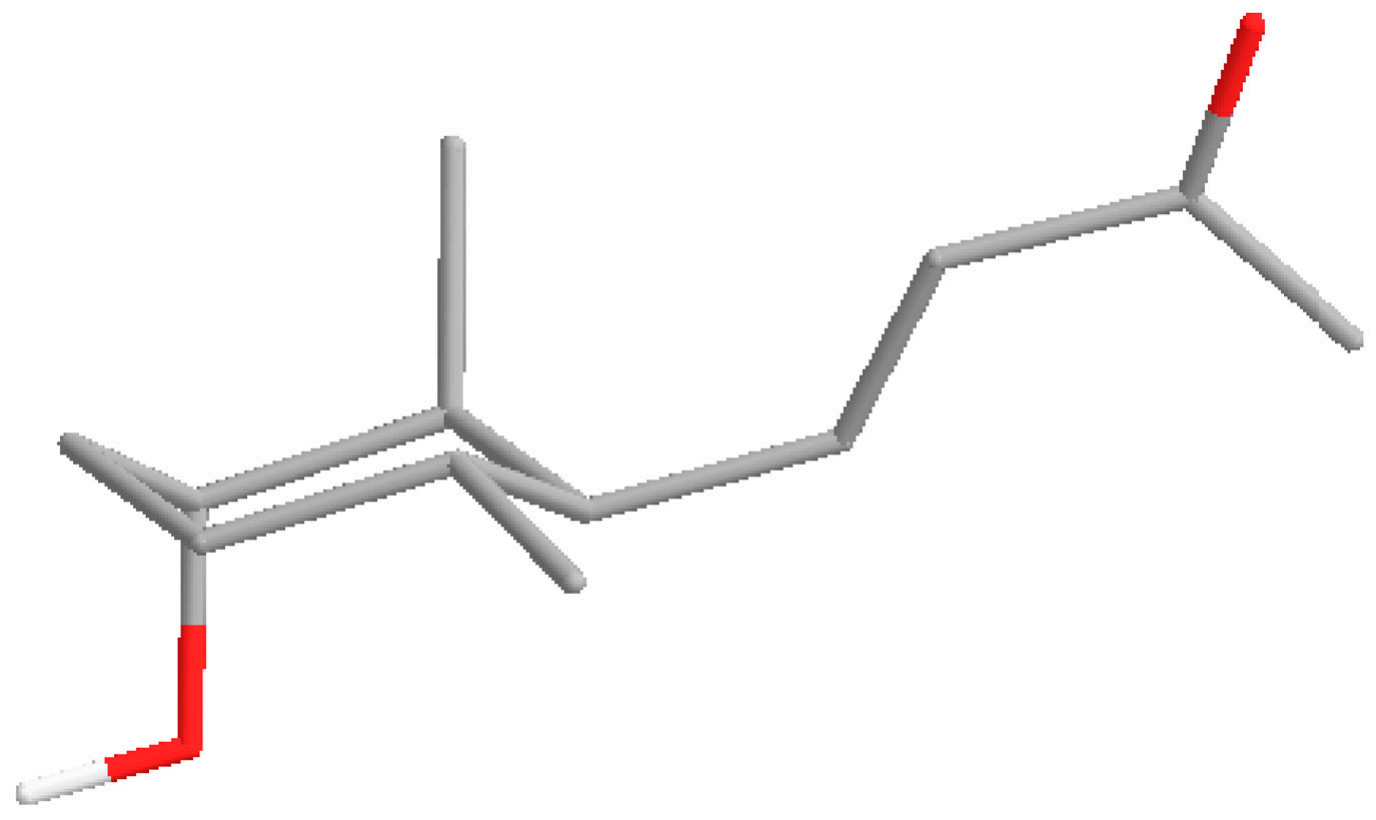	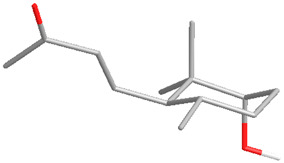

**Table 2:** Calculated conformers of compound **2** that constitute more than 95% of the conformations’ population.

Conf. No.	Relative Energy (kJ/mol)	Boltzmann Weights	Cumulative Boltzmann Weights	Compound 2 (α-OH)	Compound 2 (β-OH)
				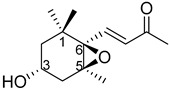	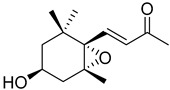
1	0.00	0.319	0.319	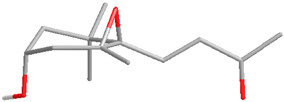	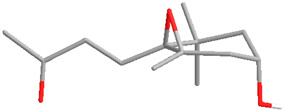
2	1.15	0.201	0.520	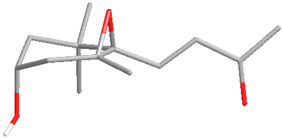	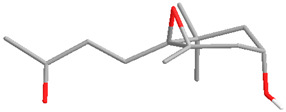
3	3.19	0.088	0.608	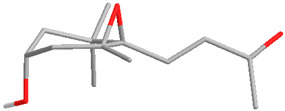	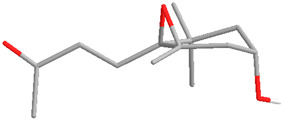
4	3.87	0.067	0.675	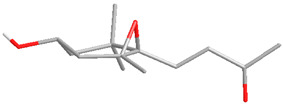	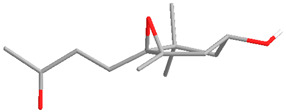
5	3.91	0.066	0.740	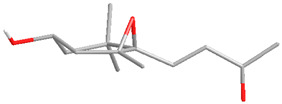	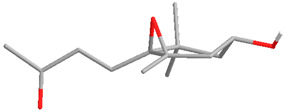
6	4.69	0.048	0.788	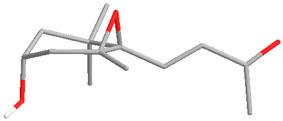	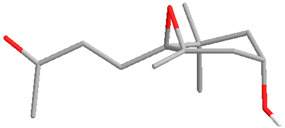
7	4.78	0.046	0.835	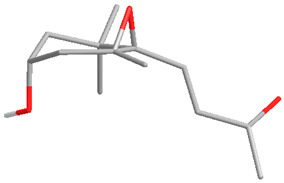	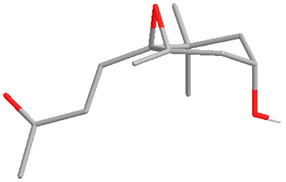
8	5.08	0.041	0.876	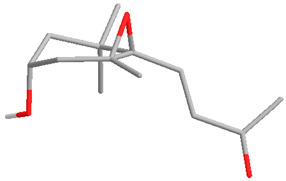	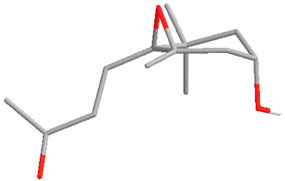
9	6.15	0.027	0.902	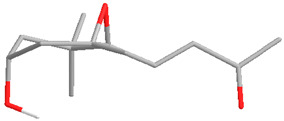	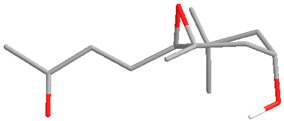
10	6.18	0.026	0.929	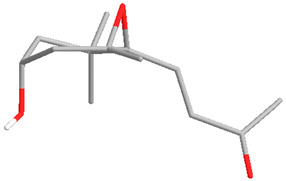	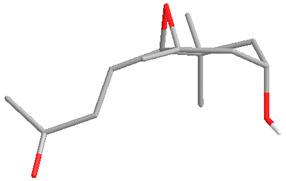
11	7.09	0.018	0.947	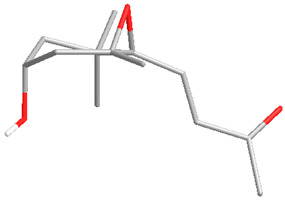	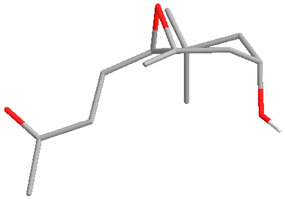
12	7.50	0.015	0.963	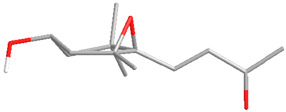	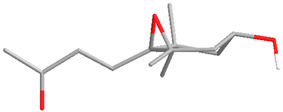

## Data Availability

The original contributions presented in the study are included in the article and Supplementary Materials.

## Supplementary Materials

The following supporting information can be downloaded at: https://www.mdpi.com/article/10.3390/metabo13121202/s1, Table S1. NMR data of compounds **1** and **2**; Figure S1. ^1^H-NMR Spectrum of Compound **1** (500 MHz, CD_3_OD); Figure S2.^13^C-NMR Spectrum of Compound **1** (125 MHz, CD_3_OD); Figure S3. DEPT Spectrum of Compound **1** in CD_3_OD; Figure S4. COSY Spectrum of Compound **1** (500 MHz, CD_3_OD); Figure S5. HMQC Spectrum of Compound **1** (500 MHz, CD_3_OD); Figure S6. HMBC Spectrum of Compound **1** (500 MHz, CD3OD); Figure S7. NOESY Spectrum of Compound **1** (500 MHz, CD3OD); Figure S8. HRESI-MS of Compound **1**; Figure S9. ^1^H-NMR Spectrum of Compound **2** (500 MHz, CD_3_OD); Figure S10. ^13^C-NMR Spectrum of Compound **2** (125 MHz, CD_3_OD); Figure S11. TOCSY Spectrum of Compound **2** (500 MHz, CD_3_OD); Figure S12. HMQC Spectrum of Compound **2** (500 MHz, CD_3_OD); Figure S13. HMBC Spectrum of Compound **2** (500 MHz, CD_3_OD); Figure S14. NOESY Spectrum of Compound **2** (500 MHz, CD_3_OD); Figure S15. HRESI-MS of Compound **2**.
