# Supplementary material for: Tetraenone A: A New β-Ionone Derivative from Tetraena aegyptia
Source: Metabolites. 2023 Dec 18;13(12):1202. doi: 10.3390/metabo13121202 (PMC10744760; doi:10.3390/metabo13121202)
Supplement: Supplementary file 1 [file metabolites-13-01202-s001.zip › metabolites-2729723-supplementary.pdf]

metabolites

MDPI

SUPPLEMENTARY MATERIAL

Tetraenone A: A new β-ionone Derivative from *Tetraena aegyptia*

Ahmed Ashour [1,2*], Asmaa E. Sherif [1,2], Selwan M. El-Sayed[3], Ji-Young Kim [4], Dae Sik Jang [4], Abtin Anvari [5] , Abdelbasset A. Farahat [5,6], Sabrin R. M. Ibrahim [7,8] , Gamal A. Mohamed [9], Bayan E. Ainousah [10], Raghad F. Aljohani [11], Razan R. Al-Hejaili [11], Rahaf Khoja [11], Ahmed H.E. Hassan [3,*] and Ahmed A. Zaki [2,12]

1 Department of Pharmacognosy, Faculty of Pharmacy, Prince Sattam Bin Abdulaziz University, Al-kharj 11942, Saudi Arabia; ahmedadelashour@yahoo.com (A.A.); asmaasherif80@yahoo.com (A.E.S.)
2 Department of Pharmacognosy, Faculty of Pharmacy, Mansoura University, Mansoura 35516, Egypt
3 Department of Medicinal Chemistry, Faculty of Pharmacy, Mansoura University, Mansoura 35516, Egypt; selwanmahmoud@hotmail.com (S.M.E.); ahmed_hassan@mans.edu.eg (A.H.E.H.)
4 Department of Life and Nanopharmaceutical Sciences, Kyung Hee University, Seoul 02447, Republic of Korea; jk293@khu.ac.kr (J.Y.K.); dsjang@khu.ac.kr (D.S.J.)
5 Master of Pharmaceutical Sciences Program, California Northstate University, 9700 W Taron Dr., Elk Grove, CA 95757, U.S.A.; abtin.anvari8815@cnsu.edu (A.A.); abdelbasset.farahat@cnsu.edu (A.A.F.)
6 Department of Pharmaceutical Organic Chemistry, Faculty of Pharmacy, Mansoura University, Mansoura 35516, Egypt
7 Preparatory Year Program, Department of Chemistry, Batterjee Medical College, Jeddah 21442, Saudi Arabia; sabrin.ibrahim@bmc.edu.sa
8 Department of Pharmacognosy, Faculty of Pharmacy, Assiut University, Assiut 71526, Egypt; sabreen.ibrahim@pharm.aun.edu.eg
9 Department of Natural Products and Alternative Medicine, Faculty of Pharmacy, King Abdulaziz University, Jeddah 21589, Saudi Arabia; gahussein@kau.edu.sa
10 Department of Pharmaceutical Chemistry, Faculty of Pharmacy, Umm Al-Qura University, Makkah 21955, Saudi Arabia; baaunosah@uqu.edu.sa
11 College of Pharmacy, Taibah University, Medina, 42353, Saudi Arabia; Phraghad0@gmail.com (R.F.A.), razan_alhujili@outlook.sa (R.R.A.); Ph.rahafkhoja@gmail.com (R.K.)
12 Department of Medicinal Chemistry, College of Pharmacy, University of Florida, Gainesville, Florida 32610, United States; ahmedawadzaki@yahoo.co.uk
* Correspondence: ahmedadelashour@yahoo.com (A.A.); ahmed_hassan@mans.edu.eg (A.H.E.H.); sabrin.ibrahim@bmc.edu.sa

metabolites MDPI

List of tables and figures

Table S1. NMR data of compounds **1** and **2**

Figure S1. ^{1}H-NMR Spectrum of Compound **1** (500 MHz, CD3OD)

Figure S2.^{13}C-NMR Spectrum of Compound **1** (125 MHz, CD3OD)

Figure S3. DEPT Spectrum of Compound **1** in CD3OD

Figure S4. COSY Spectrum of Compound **1** (500 MHz, CD3OD)

Figure S5. HMQC Spectrum of Compound **1** (500 MHz, CD3OD)

Figure S6. HMBC Spectrum of Compound **1** (500 MHz, CD3OD)

Figure S7. NOESY Spectrum of Compound **1** (500 MHz, CD3OD)

Figure S8. HRESI-MS of Compound **1**

Figure S9. ^{1}H-NMR Spectrum of Compound **2** (500 MHz, CD3OD)

Figure S10.^{13}C-NMR Spectrum of Compound **2** (125 MHz, CD3OD)

Figure S11. TOCSY Spectrum of Compound **2** (500 MHz, CD3OD)

Figure S12. HMQC Spectrum of Compound **2** (500 MHz, CD3OD)

Figure S13. HMBC Spectrum of Compound **2** (500 MHz, CD3OD)

Figure S14. NOESY Spectrum of Compound **2** (500 MHz, CD3OD)

Figure S15. HRESI-MS of Compound **2**

Table S1. NMR data of compounds **1** and **2**

	1		2	
#	δ_C	δ_H	δ_C	δ_H
1.	36.6	-	35.4	-
2.	75.7	4.53, *tt* (11.6, 4.4)	43.3	1.48, *dd* (13.1, 9.9)
				1.79, *d* (12.4)
3.	48.1	1.31, *t* (12.1)	72.4	4.51, *m*
		2.31, *dd* (9.7, 5.2)		
4.	42.4	1.11, *m*	38.2	1.96, *dd* (14.8, 7.8)
		2.02, *m*		2.50, *dd* (14.8, 5.2)
5.	31.9	1.79, *m*	67.5	-
6.	58.7	1.61, *t* (10.6)	70.4	-
7.	151.4	6.69, *dd* (15.9, 10.3)	144.5	7.17, *d* (15.8)
8.	134.8	6.11, *d* (15.9)	133.5	6.20, *d* (15.8)
9.	200.8	-	199.7	-
10.	26.9	2.28, *s*	26.9	2.30, *s*
11.	21.6	0.99, *s*	25.1	0.98, *s*
12.	31.6	0.91, *s*	28.7	1.25, *s*
13.	21.5	0.86, *d* (6.5)	19.7	1.19, *s*

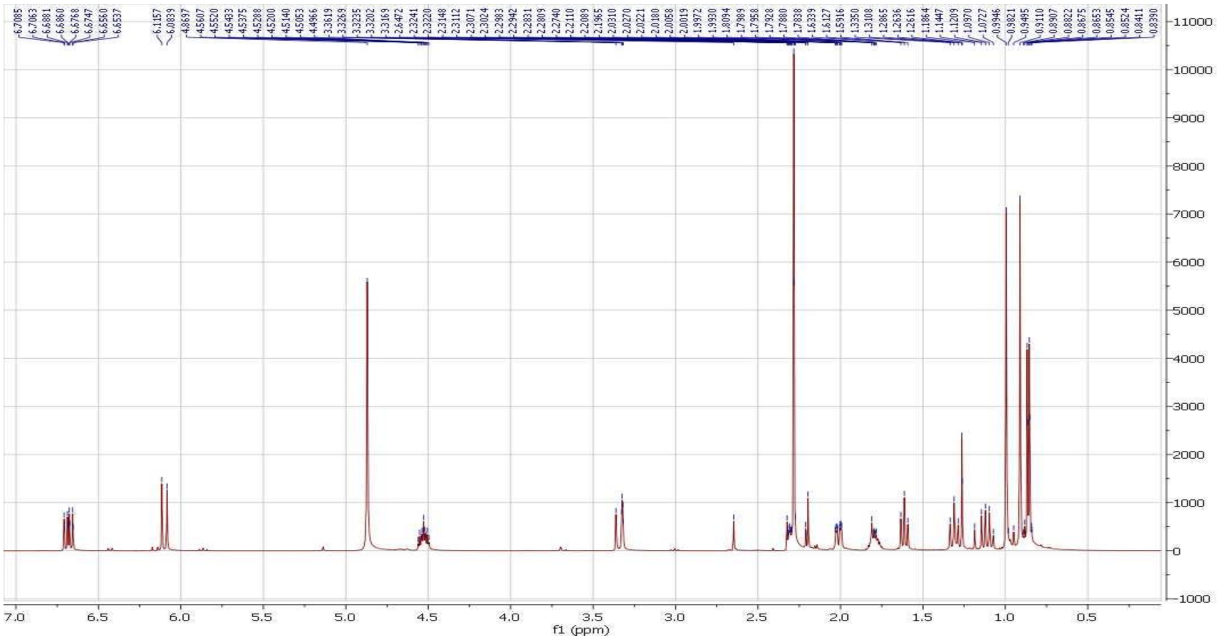

Figure S1. ^{1}H-NMR Spectrum of Compound **1** (500 MHz, CD3OD)

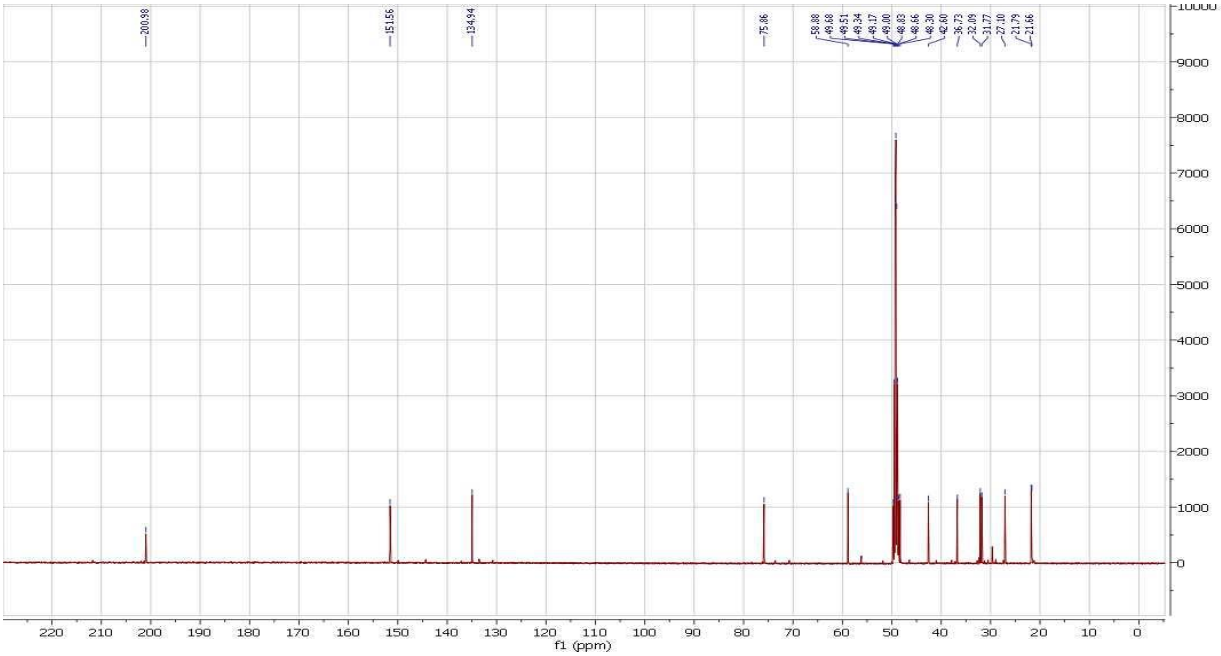

Figure S2. ^{13}C-NMR Spectrum of Compound **1** (125 MHz, CD3OD)

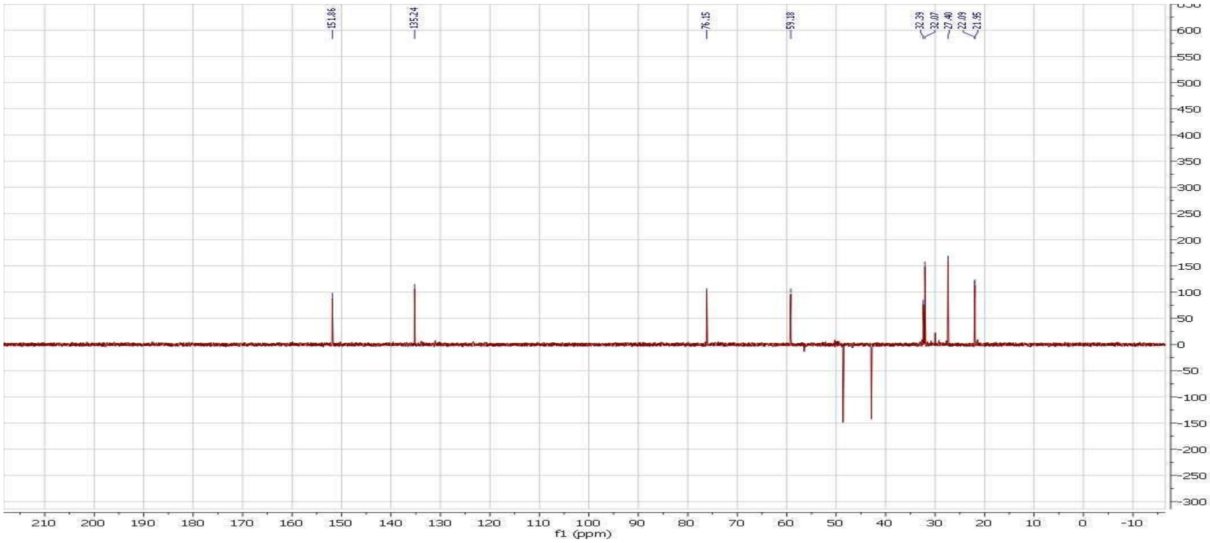

Figure S3. DEPT Spectrum of Compound **1** in CD3OD

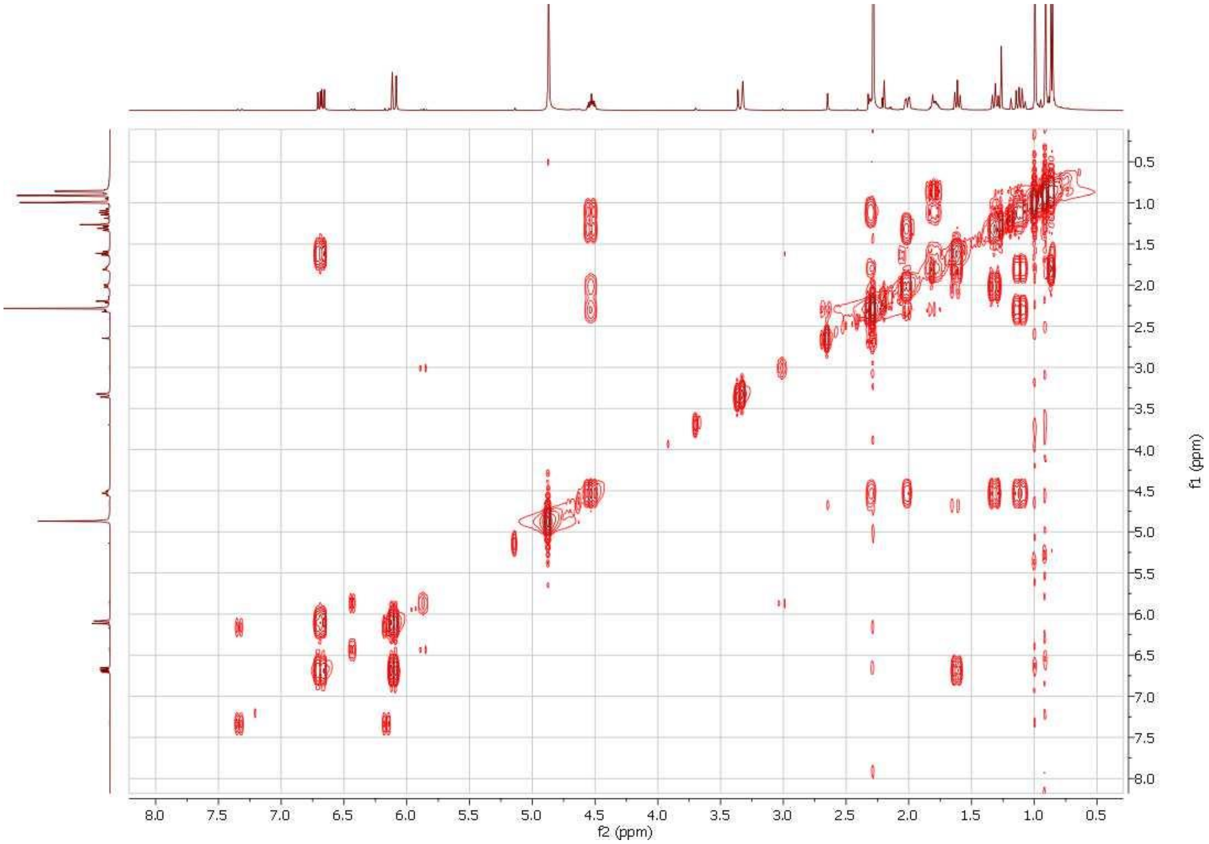

Figure S4. COSY Spectrum of Compound **1** (500 MHz, CD3OD)

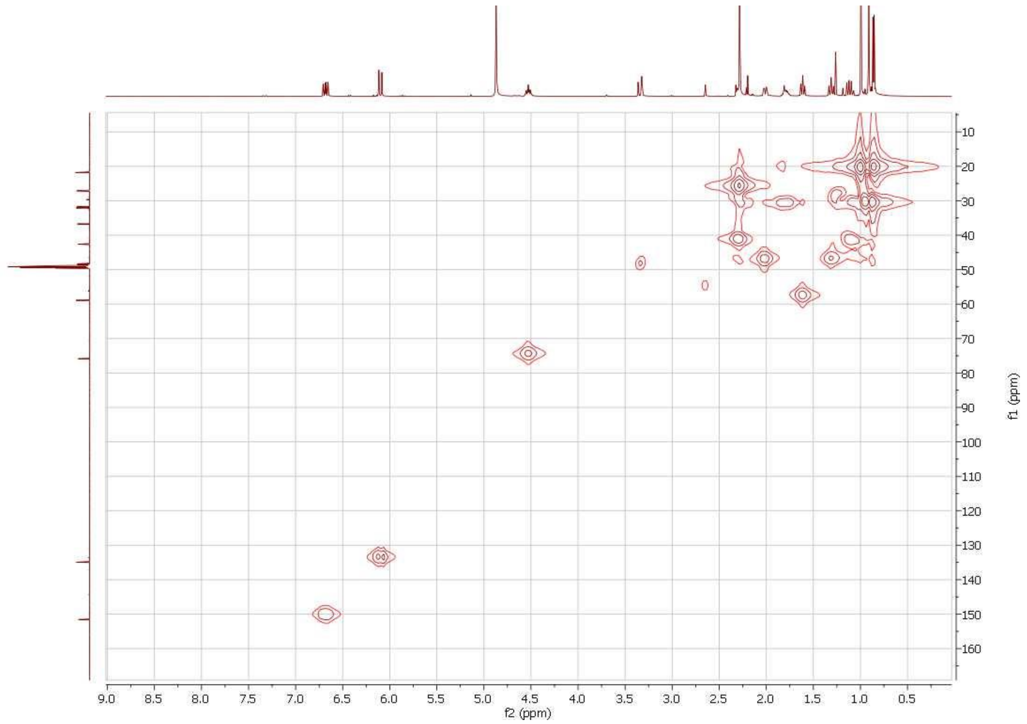

Figure S5. HMQC Spectrum of Compound **1** (500 MHz, CD3OD)

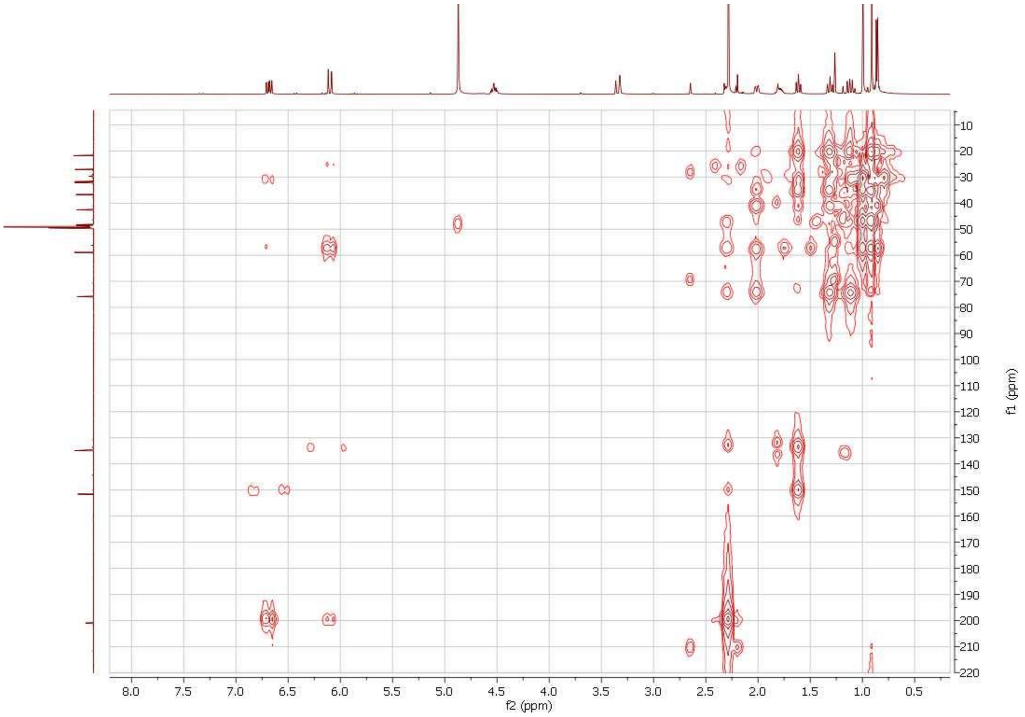

Figure S6. HMBC Spectrum of Compound **1** (500 MHz, CD3OD)

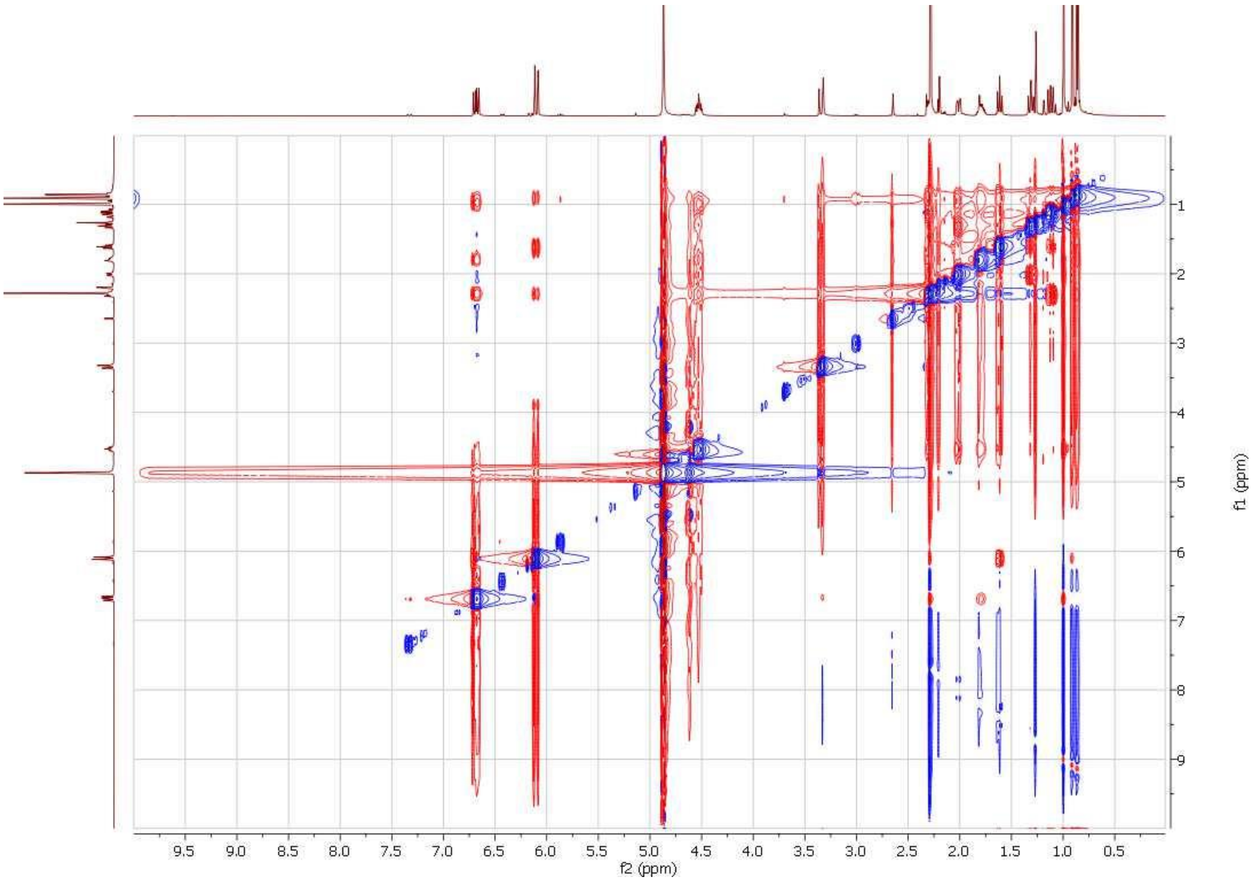

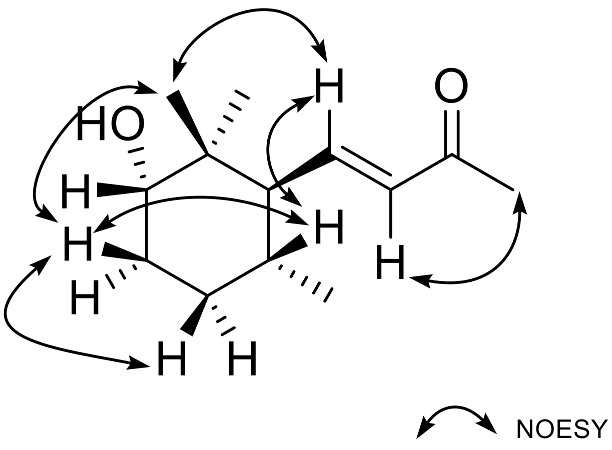

Figure S7. NOESY Spectrum of Compound **1** (500 MHz, CD3OD)

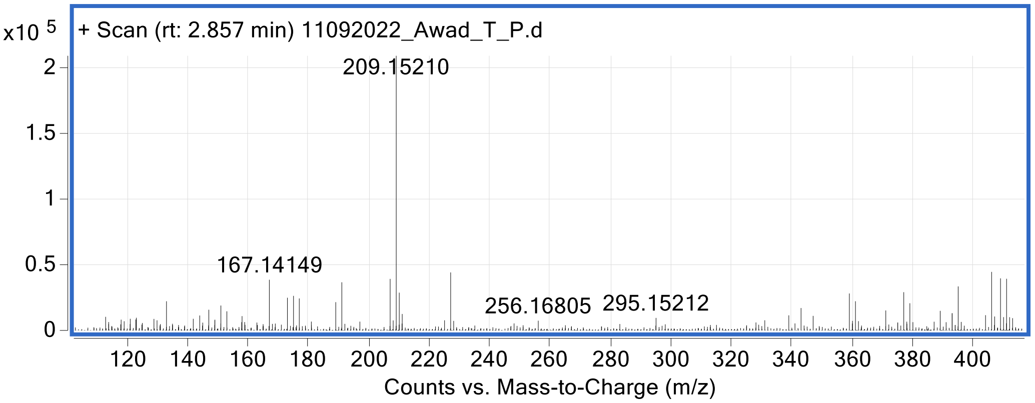

Figure S8. HRESI-MS of Compound **1**

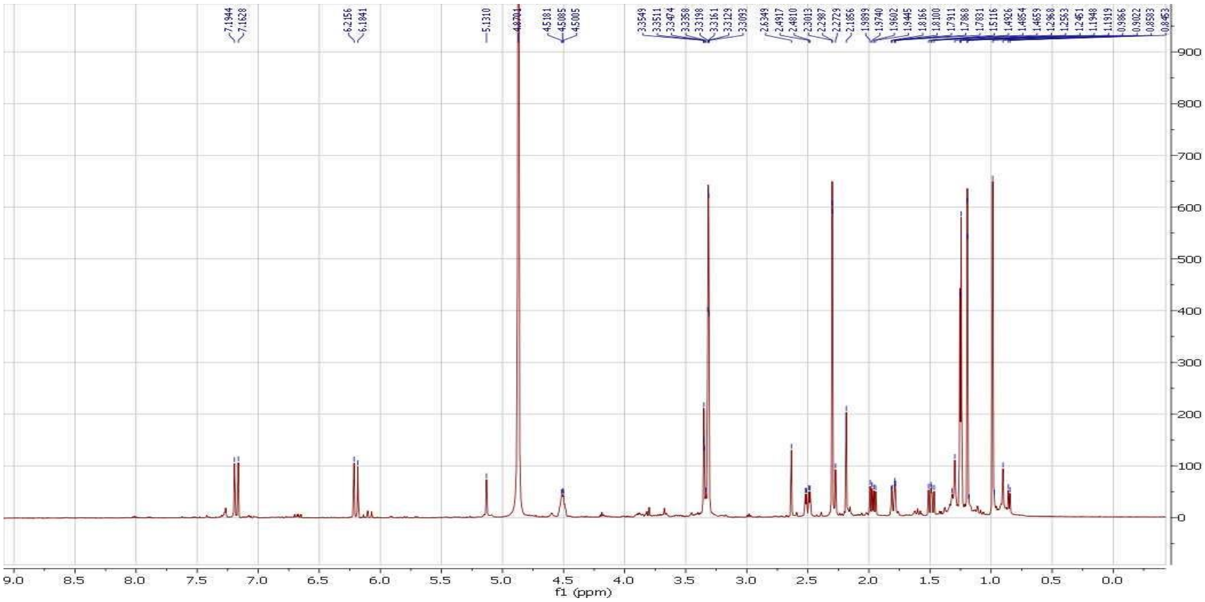

Figure S9. ^{1}H-NMR Spectrum of Compound **2** (500 MHz, CD3OD)

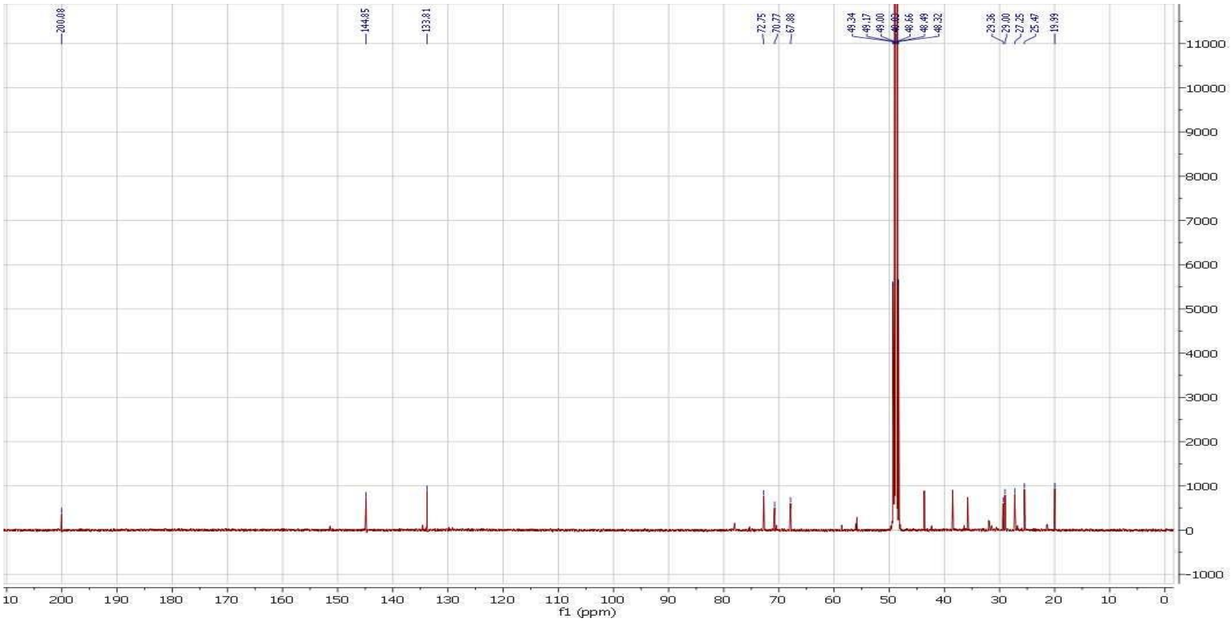

Figure S10. ^{13}C-NMR Spectrum of Compound **2** (125 MHz, CD3OD)

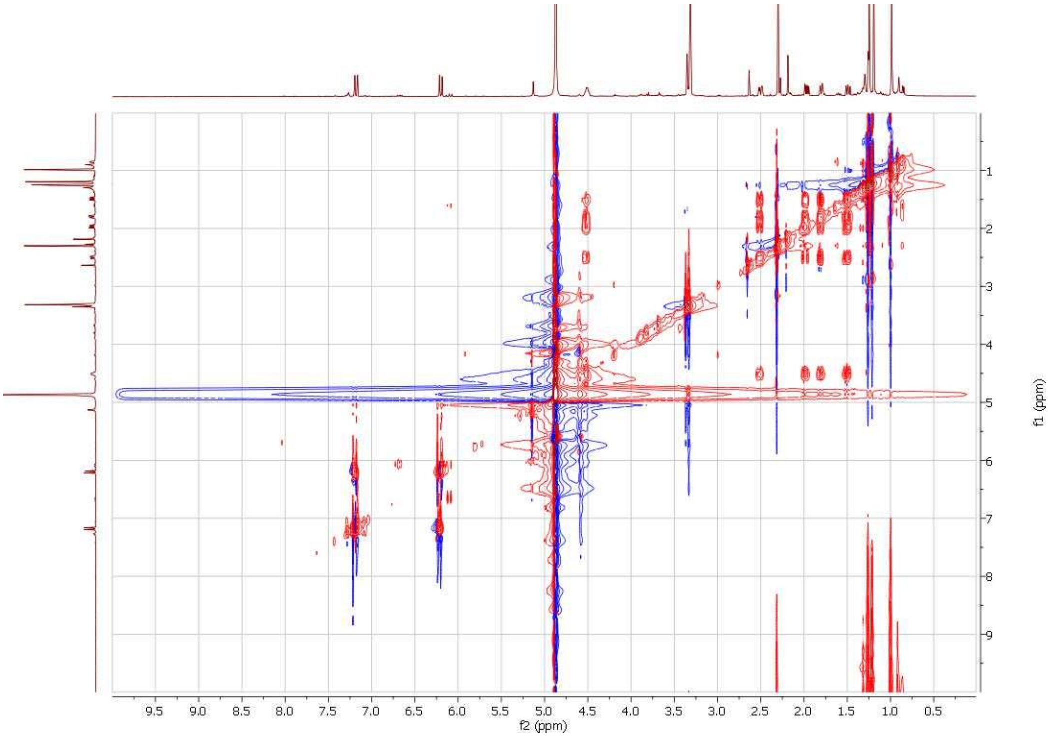

Figure S11. TOCSY Spectrum of Compound **2** (500 MHz, CD3OD)

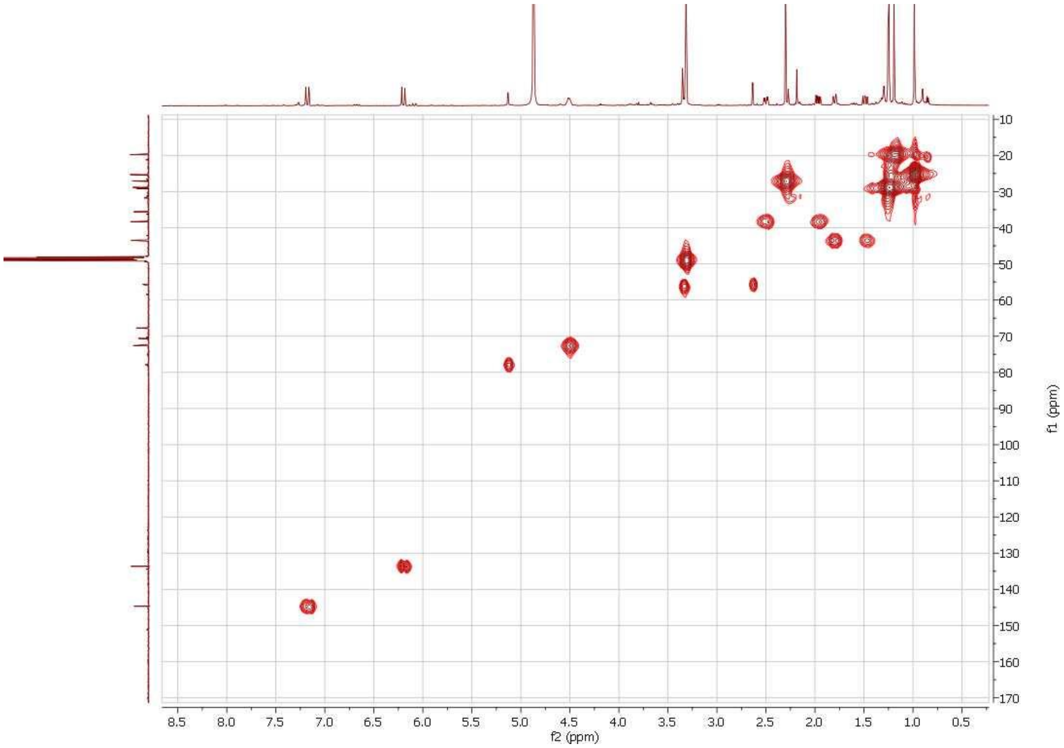

Figure S12. HMQC Spectrum of Compound **2** (500 MHz, CD3OD)

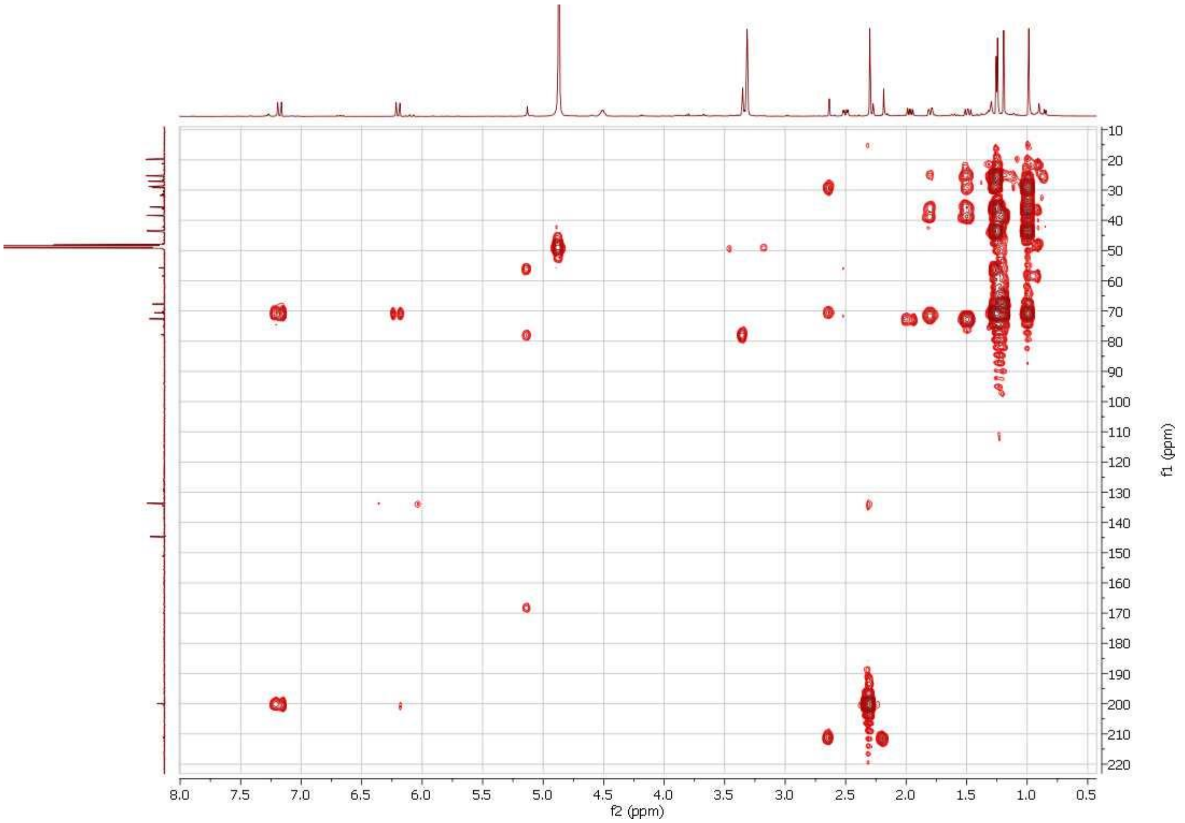

Figure S13. HMBC Spectrum of Compound **2** (500 MHz, CD3OD)

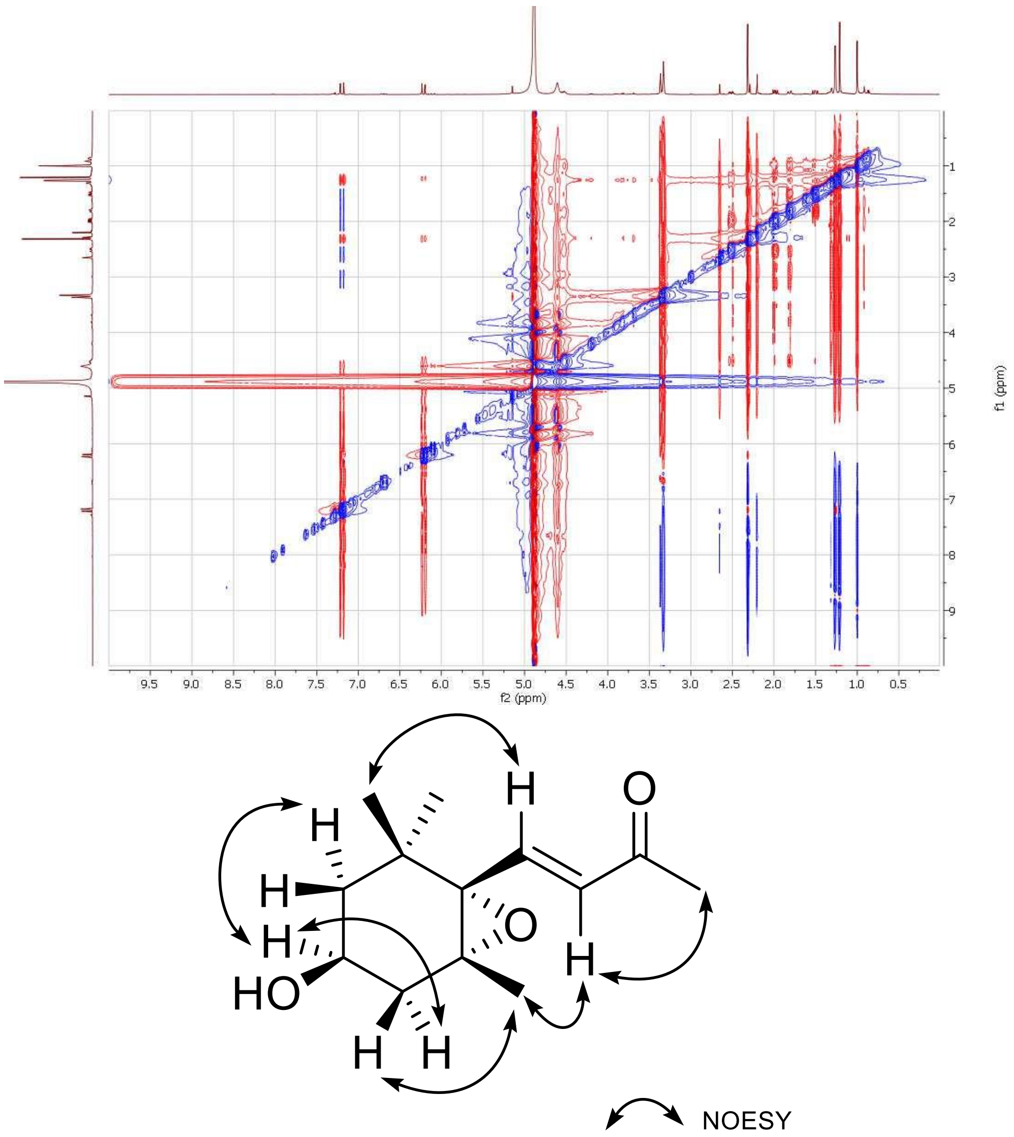

Figure S14. NOESY Spectrum of Compound **2** (500 MHz, CD3OD)

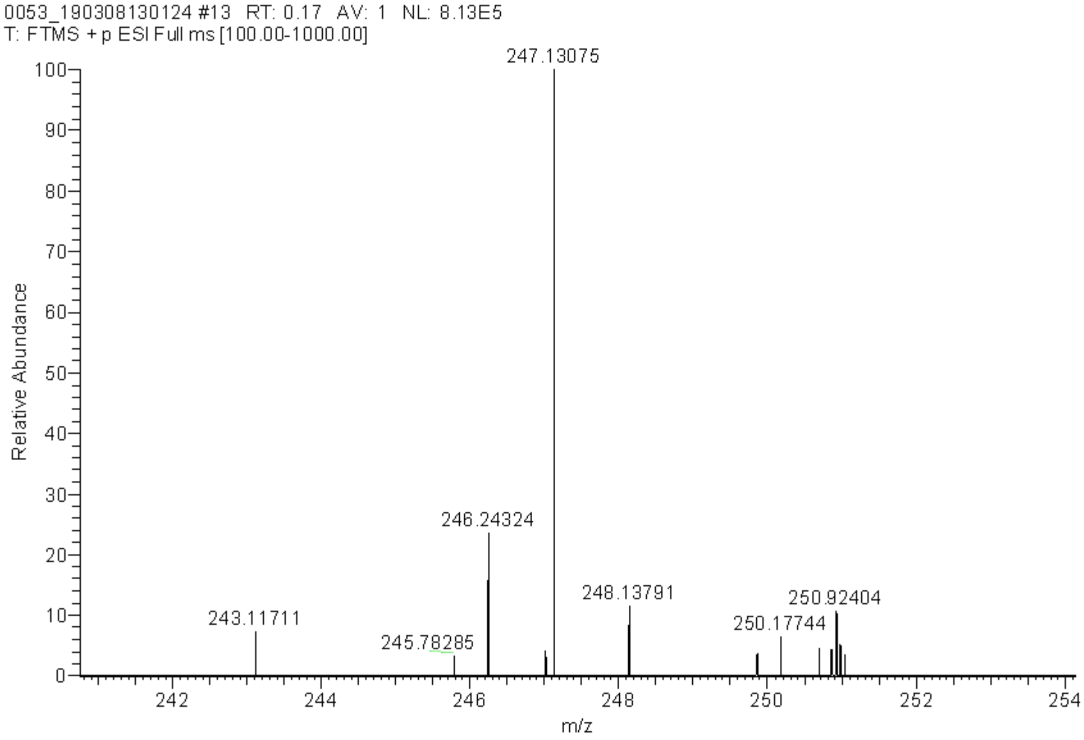

Figure S15. HRESI-MS of Compound **2**
